# Ki-67, p53 and BCL-2 Expressions and their Association with Clinical Histopathology of Breast Cancer among Women in Tanzania

**DOI:** 10.1038/s41598-019-46184-x

**Published:** 2019-07-09

**Authors:** Hidaya Mansouri, Leah F. Mnango, Erick P. Magorosa, Elingarami Sauli, Emmanuel A. Mpolya

**Affiliations:** 1 Department of Global Health and Bio-Medical Sciences, Nelson Mandela African Institution of Science and Technology, P.O. Box 447, Arusha, Tanzania; 2 Central Pathology Laboratory, Muhimbili National Hospital, P.O. Box 65002, Dar es Salaam, Tanzania; 3 Centre for Research, Agricultural Advancement, Teaching Excellence and Sustainability in Food and Nutrition Security (CREATES-FNS), Nelson Mandela African Institution of Science and Technology, P.O. Box 447, Arusha, Tanzania

**Keywords:** Cancer, Prognostic markers

## Abstract

This study associated Ki-67, p53, and BCL-2 markers with clinical histopathological (CH) features using currently available limited data on these markers in Tanzania. Retrospective chart review study was conducted among females with confirmed breast cancer (BC) at Muhimbili National Hospital in Tanzania between 2016 and 2017. Inclusion criteria were met by 76 patients with a mean age of 51.32 ± 14.28 years. Of these, 86.4% were stage III and IV, whereas 83.5% cases had grade 2 and grade 3. Upon immunostaining, 85.5% and 57.9% were Ki-67 and BCL-2 positive respectively. Log-linear analysis showed no statistically significant association among biomarkers expression and CH features. However, multinomial linear regression showed higher possibility for association between high expression of Ki-67, low expression of p53 and high expression of BCL-2 with age, grade, stage and tumor (T) stage. BCL-2 was positively correlated with Ki-67 expression contrary to p53, which was negatively correlated with BCL-2. Conclusively, there is evidence of correlation between the studied markers with CH features. However, studies with larger sample sizes will likely reveal significant associations that will validate the role of these markers as tools for evaluating treatment response in individualized therapeutic schemes in Tanzania.

## Introduction

Accurate and new methods for early breast cancer screening and treatment monitoring need to be developed and applied in low- and middle-income countries like Tanzania, where scarcity of health facilities is common. Breast cancer (BC) is one of the non-communicable diseases (NCDs) that has become a major public health issue, taking hundreds of thousands of lives each year, especially in Sub-Saharan Africa^1–3^.

Globally, cancer is a leading cause of death, accounting for 9.6 million deaths in 2018. The most common causes of cancer deaths are lung cancer, estimated at 1.76 million deaths, colorectal cancer, with 862 000 deaths and stomach cancer with 783 000 deaths, followed by liver cancer with 782 000 deaths, and BC with 627 000 deaths, accounting for about 15% of all cancer deaths among women^4^.

In Tanzania, BC incidence is reported to be 11%, a lower rate than the average global burden, but still ranks at number two of the main causes of death among women^3^. This is subsequently due to many factors, such as environmental factors, hormonal receptor expression and genetic factors^5–7^. Several studies have reported that BC is often triggered by an over-expression of biomarkers that are most commonly defined by estrogen-receptor (ER), progesterone receptor (PR), and Human Epidermal growth factor Receptor 2 (HER2) status^5,8,9^. These markers have been demonstrated to be important prognostic factors for endocrine therapy, which is among the common treatment methods available in Tanzania.

In BC pathogenesis, previous studies have shown that it results from mutations and abnormal changes in the genes responsible for cellular growth regulation, homeostasis, and maintenance of health^10,11^. Novel molecular markers, such as Ki-67, p53, and BCL-2 are thus emerging as tools for classifying BCs, guiding therapy, and predicting treatment response and prognosis^12,13^. Unfortunately, testing for these biomarkers is not routinely performed in many developing countries such as Tanzania, which contributes to the scarcity of data for cancer management. Tumor markers have shown to be prominent tools for determining prognosis and informing treatment plans^13^. The current study therefore aimed at investigating the relationship between Ki-67, p53, and BCL-2 expression with clinical and histopathological factors in patients with BC.

## Results

**Figure 1:**
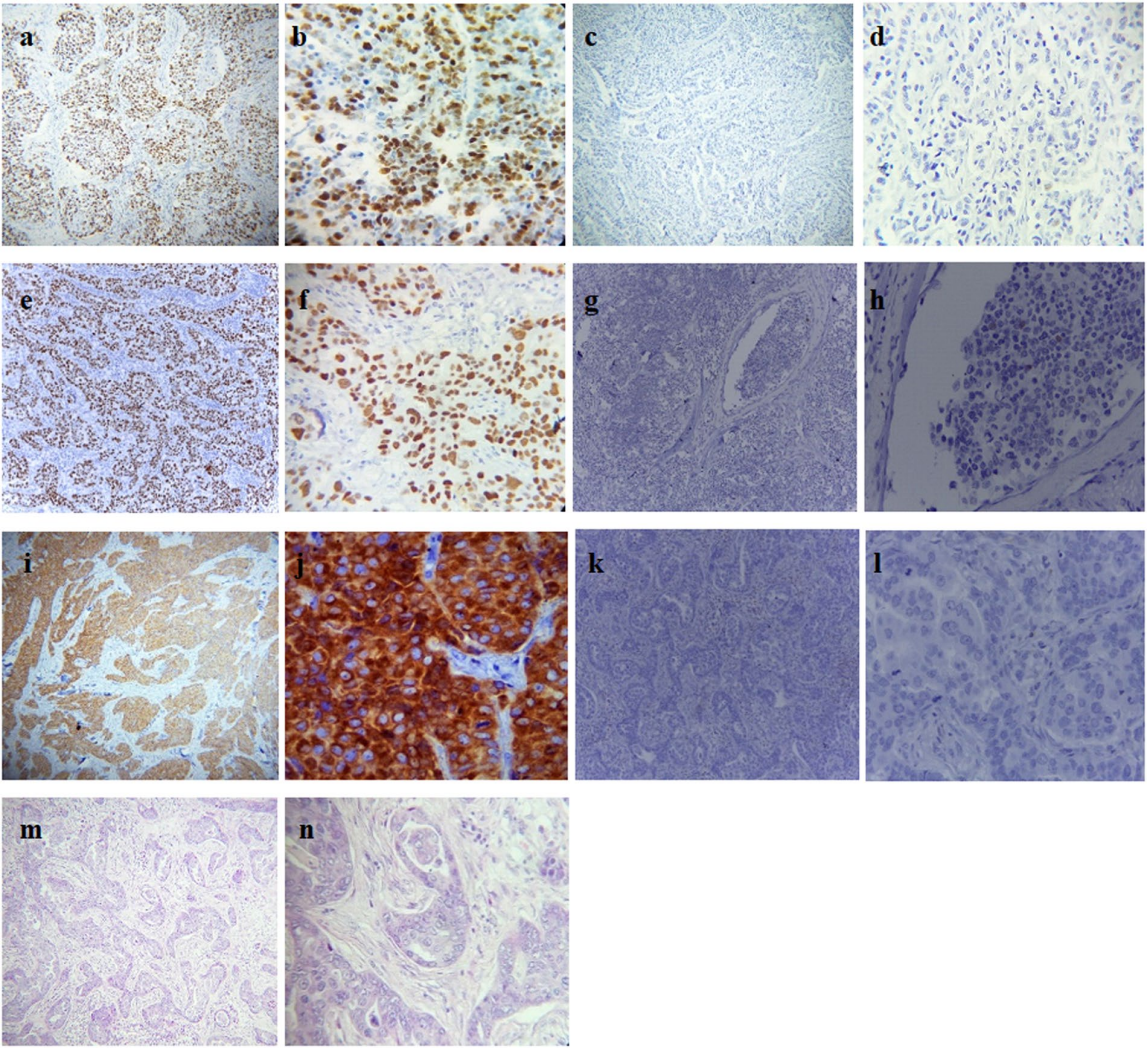
Monograghs. (**a** and **b**) Nuclear positively stained for Ki-67 at 10x and 40x *hpf respectively; (**c** and **d**) Nuclear negatively stained for Ki-67 at 10x and 40x hpf respectively; (**e** and **f**) Nuclear positively stained for p53 at 10x and 40x hpf respectively; (**g** and **h**) Nuclear negatively stained for p53 at 10x and 40x hpf respectively; (**i** and **j**) Nuclear membrane positively stained for BCL-2 at 10x and 40x hpf respectively: (**k** and **l**) nuclear membrane negatively stained for BCL-2 at 10x and 40x hpf respectively; (**m** and **n**) H&E staining for infiltrating ductal carcinoma (IDC) at 10x and 40x hpf respectively. *hpf: High-power field.

Retrieved blocks from patients with confirmed breast carcinoma, clinical histopathological information, and good histological criteria (spatial arrangement of the cells, morphometric characteristics of the nuclei, tubules formation, and number of cancer dividing cells) were selected. Out of 775 cases recorded as in or outpatients, 391 (49.4%) were malignant. However, only 76 (9.6%) cases had histological criteria and complete information, and thus qualified for the Hematoxylin & Eosin (H&E) slide review and subsequent biomarker analysis. The 76 samples were analyzed for Ki-67, p53 and BCL-2 biomarkers via immunohistochemistry (IHC), followed by subsequent statistical analysis (Fig. 1).

### Clinical histopathological information and biomarker expression in selected patients

The age of diagnosed patients ranged from 23 to 92 years old, with a mean age of 51.32 ± 14.28 years. Their median age (IQR) was 50 (42–61) years. Out of 76 patients classified for breast carcinoma, 74 out of 76 samples were pathologically staged, in which 3 (4.1%) and 7 (9.5%) had stages I and II, respectively at the initial time of diagnosis, in contrast to 36 (48.6%) and 28 (37.8%) who presented late stages III and IV respectively, with a tumor thickness of >2 cm. Moreover, 36 (49.3%) had mostly intermediate grade (grade 2), followed by 25 (34.2%) of high grade (grade 3) breast carcinoma (Table 1). Clinico-histopathological findings revealed that the majority of patients 66 (86.8%) presented with infiltrating ductal carcinoma (IDC), followed by 5 (6.6%) of lobular carcinoma (ILB), 1 (1.3%) of medullary carcinoma (MC), 1 (1.3%) of mucinous carcinoma (MucC) and 3 (3.9%) with others.

**Table 1:** Patients and clinico-histopathological characteristics.

Characteristic	All (n = 76)
**Age** (**years**)	51.32 ± 14.28
**T stages**	
T1	6 (8.10%)
T2	14 (18.91%)
T3	18 (24.32%)
T4	36 (48.64%)
Missing cases	2
**Nodule status**	
N0	13 (17.6%)
N1	10 (13.5%)
N2	30 (40.5%)
N3	15 (20.3%)
Nx	6 (7.4%)
Missing cases	2
**Grade**	
Grade 1	12 (16.4%)
Grade 2	36 (49.3%)
Grade 3	25 (34.2%)
Missing cases	3
**Stage**	
Stage I	3 (4.1%)
Stage II	7 (9.5%)
Stage III	36 (48.6%)
Stage IV	28 (37.8%)
Missing cases	2
**Histologic subtype**	
IDC	66(86.8%)
ILC	5 (6.6%)
MC	1 (1.3%)
MucC	1 (1.3%)
Others	3 (3.9%)
**Biomarkers expression**	
Ki-67 (High)	65 (85.5%)
(Low)	11 (14.5%)
p53 (High)	30 (39.5%)
(Low)	46 (60.5%)
BCL-2 (High)	44 (57.9%)
(Low)	11 (42.1%)

**Figure 2:**
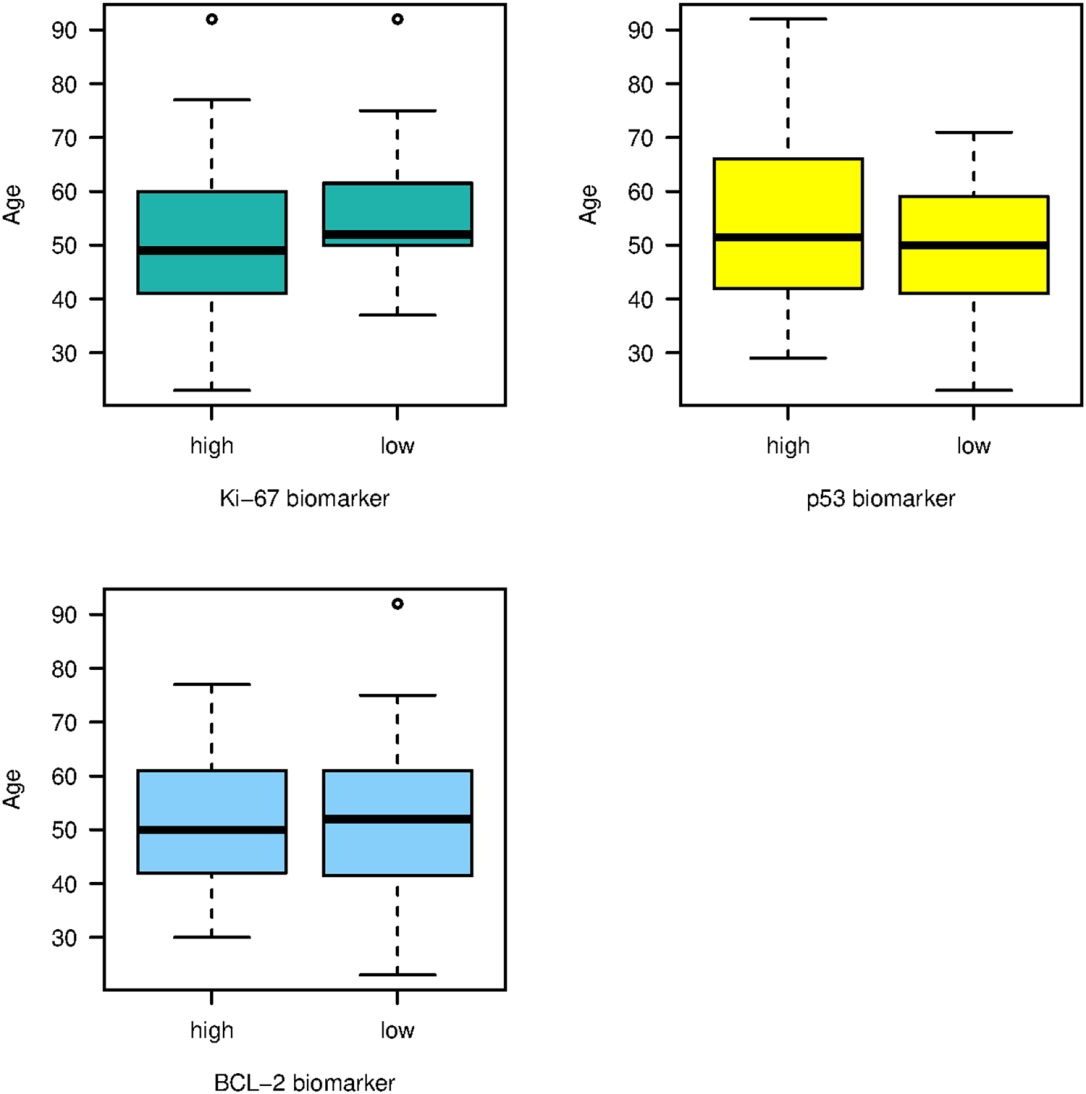
Distribution of Ki-67, p53 and BCL-2 among age-groups.

Furthermore, immunostaining revealed that most of the patients with invasive breast carcinoma (IBC) strongly expressed Ki-67 (Ki-67+) and BCL-2 (BCL-2+), with proportions of 85.5% (65/76) and 57.9% (44/76) respectively. In contrast, a large number of cases, 46 (60.5% of patients), expressed a negative response to p53 staining (Table 1). Notably, positive biomarkers expression was mostly found in premenopausal patients (<50 years old) (Fig. 2).

### The relationship between clinico-histopathological characteristics with biomarkers expression

**Figure 3:**
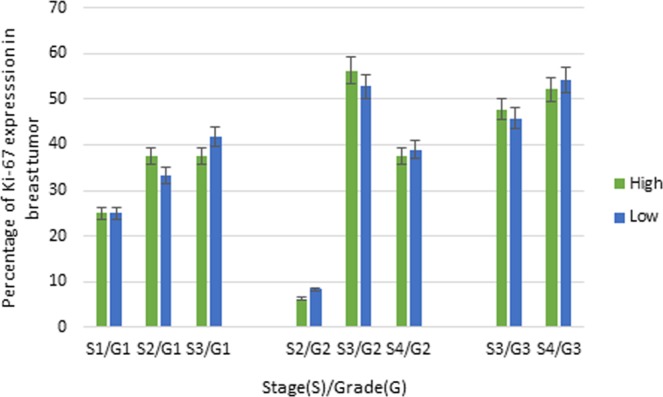
Association between Ki-67 with clinical and histopathological grades.

**Figure 4:**
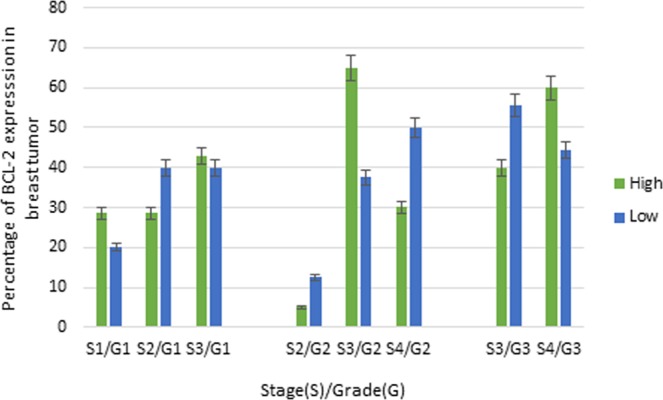
Association between BCL-2 with clinical and histopathological grades.

The current findings showed that Ki-67 and BCL-2 biomarkers were highly expressed in stage III and/or IV and grade 2 and/or grade 3 of breast carcinoma (Figs 3 and 4). Likewise, Ki-67 was marginally statistically significantly associated with histopathological grade (Fisher’s exact test, p-value = 0.054). In contrast, high expression of p53 showed significant association with primary tumor (Fisher’s exact test, p-value = 0.007). Moreover, there was no significant association between p53 expression and histopathological grading (p-value = 0.560) and clinical staging (p-value = 0.121), as well as lymph node status (p-value = 0.209). Furthermore, there was no statistically significant association between the expression of BCL-2 with the status of primary tumor (T) (p-value = 0.419) and lymph node (p-value = 0.939). Additionally, no dependence was observed between biomarker expressions with age and histological subtype, meaning that these biomarkers cannot be potentially used as prognostic tools across all age groups, as well as histological subtypes (Table 2).

**Table 2:** Relationship between clinic-histopathological factors with Ki-67, p53 and BCL-2 biomarkers.

	All (n = 76)	Ki-67^a^ No. (%)	p53^b^ No. (%)	BCL-2^c^ No. (%)	Fisher’s Exact test *p-*value
Age (years)*	51.32 ± 14.28	50.2 ± 14.0	54.1 ± 16.8	51.0 ± 12.5	0.167^a^
					0.428^b^
					0.411^c^
**T stage**					
T1	6 (8.10%)	3 (4.7%)	1 (3.4%)	3 (4.05%)	0.078^a^
T2	14 (18.91%)	12 (18.8%)	1 (3.4%)	9 (12.15%)	**0.007** ^**b**^
T3	18 (24.32%)	16 (25.0%)	7 (24.1%)	8 (10.80%)	0.419^c^
T4	36 (48.64%)	33 (51.5%)	20 (69.0%)	24 (32.42%)	
**Nodule status**					
N0	13 (17.6%)	11 (17.2%)	3 (10.3%)	8 (18.2%)	
N1	10 (13.5%)	8 (12.5%)	3 (10.3%)	7(15.9%)	0.338^a^
N2	30 (40.5%)	28 (43.8%)	15 (51.7%)	17 (38.6%)	0.209^b^
N3	15 (20.3%)	13 (20.3%)	4 (10.3%)	8 (18.2%)	0.939^c^
Nx	6 (7.4%)	4 (6.3%)	4 (10.3%)	4 (9.1%)	
**Grade**					
Grade 1	12 (16.4%)	8 (12.5%)	3 (10.4%)	7 (16.7%)	**0.054** ^**a**^
Grade 2	36 (49.3%)	32 (50.0%)	15 (51.7%)	20 (47.6%)	0.560^b^
Grade 3	25 (34.2%)	24 (37.5%)	11 (37.9%)	15 (35.7%)	0.949^c^
**Cancer stage**					
Stage I	3 (4.1%)	2 (3.1%)	0 (0%)	2 (4.5%)	
Stage II	7 (9.5%)	5 (7.8%)	1 (3.4%)	3 (6.8%)	0.217^a^
Stage III	36 (48.6%)	33 (51.6%)	13 (44.8%)	23 (52.3%)	0.121^b^
Stage IV	28 (37.8%)	24 (37.5%)	15 (51.7%)	16 (36.4%)	0.758^c^
**Histologic subtype**					
IDC	66 (86.8%)	57 (87.7%)	29 (96.7%)	38 (86.4%)	0.345
ILC	5 (6.6%)	4 (6.2%)	1 (3.3%)	4 (9.1%)	0.595
MC	1 (1.3%)	1 (1.5%)	0 (0%)	1 (2.3%)	0.361
MucC	1 (1.3%)	1 (1.5%)	0 (0%)	1 (2.3%)	
Others	3 (3.9%)	2 (3.0%)	0 (0%)	0 (0%)	

^a,b,c^Is *p*-value from the Fisher’s exact test.

^*^Mean ± SD.

Multinomial logistic regression analysis investigating the relationship between biomarkers and clinico-histopathological characteristics showed that Ki-67, p53 and BCL-2 level (p > 0.05) were not statistically significantly associated with age, cancer stage, cancer grade, T stage and nodule status. However, these biomarkers could potentially be predictors of poor outcome (Table 3) when stratified into low and high levels. The odds ratios for associating high Ki-67 expression (as compared to low Ki-67 expression) with cancer grade and tumor stage were; OR 1.254; 95% CI 0.586–2.683 and OR 1.374; 95% CI 0.705–2.677 respectively. On the other hand, the odds ratios for associating high BCL-2 expression (as compared to low BCL-2 expression) with cancer grade and tumor stage were; OR 1.170; 95% CI 0.512–2.676 and OR 1.533; 95% CI 0.746–3.149 respectively. Furthermore, with regards to high p53 expression, the odds ratios for associating it with tumor stage were; OR 1.967; 95% CI 0.834–4.627 while with low p53 expression, the odds ratios for associating it with cancer grade were; OR 1.236; 95% CI 0.550–2.779. While these odds ratios do contain a null value of 1, which could be because of our smaller sample size (limited by data and inclusion criteria), we still think that these results do indicate a potential promise for these biomarkers to be confirmed in high-powered studies, as tools for evaluating treatment response in individualized therapeutic schemes.

**Table 3:** Multinomial logistic regression analysis predicting outcomes.

Low BCL-2^a^		B	Std. Error	Wald	P value.	OR (Odd Ratio)	95% Confidence Interval for OR	
							Lower Bound	Upper Bound
High Ki-67	Age	−0.008	0.017	0.2356	0.627	**0**.991*	0.958	1.025
	Cancer stage	−0.344	0.527	0.4279	0.512	0.708	0.252	1.990
	Cancer grade	0.226	0.388	0.3406	0.559	**1**.254*	0.586	2.683
	T stage	0.318	0.340	0.8763	0.349	**1**.374*	0.705	2.677
	Nodule status	−0.089	0.233	0.1463	0.702	0.914	0.579	1.444
	Histologic subtype	−0.224	0.241	0.8666	0.351	0.798	0.497	1.282
Low Ki-67	Age	0.048	0.030	2.5518	0.110	1.049	0.989	1.114
	Cancer stage	−0.275	0.853	0.1042	0.7467	0.759	0.142	4.041
	Cancer grade	−0.854	0.740	1.3330	0.248	0.425	0.099	1.815
	T stage	−0.113	0.536	0.0444	0.833	0.893	0.312	2.554
	Nodule status	0.167	0.404	0.1715	0.678	**1**.182*	0.535	2.609
	Histologic subtype	−0.025	0.371	0.0048	0.944	0.974	0.470	2.020
High p53	Age	0.009	0.019	0.2358	0.627	1.009	0.970	1.050
	Cancer stage	−0.079	0.626	0.0161	0.898	0.923	0.270	3.154
	Cancer grade	0.002	0.484	1.8117E-05	0.996	1.002	0.387	2.588
	T stage	0.675	0.436	2.3904	0.122	**1**.965*	0.834	4.627
	Nodule status	0.011	0.271	0.0018	0.965	1.011	0.594	1.720
	Histologic subtype	−0.730	0.694	1.1066	0.292	0.481	0.123	1.878
Low p53	Age	−0.006	0.018	0.1446	0.703	0.993	0.958	1.029
	Cancer stage	−0.495	0.561	0.7800	0.377	0.609	0.202	1.829
	Cancer grade	0.2121	0.413	0.2651	0.606	**1**.236*	0.550	2.779
	T stage	0.065	0.356	0.03384	0.853	1.067	0.531	2.146
	Nodule status	−0.063	0.252	0.0627	0.802	0.938	0.572	1.540
	Histologic subtype	−0.145	0.230	0.3980	0.528	0.864	0.550	1.358
High BCL-2	Age	−0.001	0.018	0.0054	0.941	0.998	0.963	1.035
	Cancer stage	−0.542	0.559	0.9396	0.332	0.581	0.194	1.740
	Cancer grade	0.157	0.421	0.1394	0.708	**1**.170*	0.512	2.676
	T stage	0.427	0.367	1.3573	0.243	**1**.533*	0.746	3.149
	Nodule status	−0.071	0.249	0.0821	0.774	0.930	0.570	1.518
	Histologic subtype	−0.456	0.326	1.9561	0.161	0.633	0.334	1.200

^a^The reference category.

*OR >1 has causative effect on the cancer patients.

### Antagonistic expression among Ki-67, p53 and BCL-2

During the post-analytical microscopic phase, it was interesting to find out that Ki-67 and p53 were antagonistically expressed when viewed in combination with BCL-2. When statistically tested, correlation test for p53 showed significant negative correlation with BCL-2 expression (Correlation coefficient = −0.238, p-value = 0.038), whereas, BCL-2 expression was significantly positively correlated with Ki-67 expression (Correlation coefficient = 0.331, p-value = 0.004). However, no significant correlation was observed between Ki-67 and p53 expressions. These findings are further corroborated by a correlation matrix in Table 4, which shows that despite there being weak correlations, the directions of those correlations could point to something significant. We observed that the Ki-67 biomarker correlates weakly but negatively with cancer grade, while p53 biomarker correlates weakly but negatively with cancer stage. Finally, BCL-2 expression correlates positively with Ki-67 expression and negatively with p53 expression.

**Table 4:** Antagonistic biomarkers expression: *Correlation among biomarkers expression and clinico-histopathological features*.

		1	2	3	4	5	6	7	8
1.	Age								
2.	Cancer stage	0.043							
3.	Cancer grade	−0.048	0.475**						
4.	Nodule status	0.002	0.418**	0.271*					
5.	Ki-67 biomarker	0.160	−0.064	−0.268*	0.035				
6.	p53 biomarker	−0.134	−0.281*	−0.109	−0.131	0.103			
7.	BCL-2 biomarker	0.004	0.002	−0.024	0.048	0.331**	−0.238*		
8.	Tumor size	0.077	0.612**	0.337**	0.115	−0.217	−0.371**	−0.103	

^**^p-value < 0.01.

^*^p-value < 0.05.

## Discussion

As a heterogeneous disease with various biomarker expressions, one of the major challenges in breast cancer is to identify patients who will or will not benefit from appropriate treatment based on the understanding of key markers involved in BC pathogenesis. Thus, Ki-67, p53 and BCL-2 expression levels were previously reported in several studies on BC and classified as Luminal A or Luminal B subtypes^14,15^. In addition to this classification, most of Tanzanian patients attending MNH with breast cancer were tested for hormonal receptors (ER and PR), as well as HER-2, followed by a general application of endocrine therapy through Tamoxifen or Trastuzumab at Ocean Road Cancer Institute (ORCI)^8,16^. This hormonal treatment was conducted without any complementary biomarkers investigation, which could potentially counter drug resistance and treatment nonresponses.

This current study involved a relatively small number of patients with BC, and findings showed that the majority of study subjects had high expression of Ki-67 and p53, which then could further be classified as luminal B rather than luminal A when considering reported classification categories. These findings agree with research conducted by Fulga, 2017, demonstrating the molecular subtypes Ki-67 activity and p53 evolution during BC progression. Besides, in the tumorigenesis process, p53 is known as a protein responsible for DNA repair activation, apoptosis and cell growth control, by holding the cell cycle at G1 and S regulation point. Previous findings have revealed the association between Ki-67 and p53 as well as its molecular classification in luminal B subtype^17^.

Ki-67 and BCL-2 expression was mostly observed in invasive breast carcinoma (IBC) and premenopausal patients (<50 years old) in this study, probably due to their proliferative and anti-apoptotic effect as well as an up-regulation related to higher hormonal levels, and hence higher receptors expression during this age period^13,14,18,19^.

The relationships between Ki-67 expression, histopathological grading and BCL-2 expression in BC have been described by numerous studies as molecular markers mostly associated with malignant tissues^20–25^, intermediate grade, negative/low p53 (p53-) status, high levels of BCL-2 (BCL-2) expression, suggesting to be sensitive to hormonal therapy^26,27^. However, some findings were not obvious in our study, because of non-significant statistical results that are most likely due to the low power of our study. In this study, bivariate analyses revealed only marginal statistical significance but multinomial analysis did not reveal any significance. As both Ki-67/BCL2 and p53 are involved in cell proliferation and apoptosis, they play an important role in determining tumor growth and may more accurately help to define high-risk patients. Hence, patients with high Ki-67, low p53 and high BCL-2 are more likely to be at high risk of having poor prognosis compared to patients with low BCL2 in this study, which needs to be confirmed in bigger studies as suggested in this study.

Comparing this study with Awadelkarim *et al*. 2012, Shapochka *et al*. 2013 and Angel *et al*. 2014, our findings herein diverge from these authors. While they found a statistically significant relationship between biomarker expression and clinico-histopathological features, our study found no such statistically significant association, most likely due to limited sample size with low power. Following a research study by Angel *et al*., 251 cases were investigated to establish the association between tumor size, lymph node status and immunohistochemical expression of Ki-67, p53, and BCL-2 in patients with BC. Their research demonstrated a significant association between tumor size with advanced histological grade, high cell proliferation (Ki-67 expression) and p53^20^. However, with regards to age, their study showed significant association of tumor size among women over 70 years old, which is contrary to our findings, where no statistically significant association was observed across the age groups with biomarkers. Shapochka *et al*.^26^ also reported the association between Ki-67 with tumor grade but their conclusion regarding the correlation between p53 and tumor grade was not observed in this current study. Further, Filip Čečka *et al*.^28^ showed no significant relationship between BCL-2 expression with tumor grade and primary tumors, which concurs with our findings herein. The observed correlations among clinical-histopathological features (cancer stage vs cancer grade; cancer grade vs nodule status; T stage vs cancer stage/cancer grade) reported in the current study could be explained by the fact that the larger the primary tumor the greater the number of lymph nodes that will be affected in advanced cancer stage and cancer grade^29^.

During the post-analytical microscopic phase, with respect to BCL-2, the expressions of Ki-67 and p53 appeared to be antagonistic, as BCL-2 seems to promote Ki-67 expression (Pearson r = +0.331, p-value = 0.004) and suppress p53 expression (Pearson’s r = −0.238, p-value = 0.038). No significant correlation was observed between Ki-67 and p53 expressions. As a tumor suppressor protein, p53 is a protein encoded by TP53 tumor suppressor gene found in normal tissues. Its synthesis has an inhibitory effect on cell proliferation and transformation, controlling the initiation and or regulation of DNA replication, as well as transcription and regulation of response to DNA alteration^30,31^. p53 is observed in cellular processes when activated by cellular stress responses, which include cell cycle and apoptosis. Such mechanisms could therefore explain its under-expression in many tumors, including breast cancer as perhaps the case in our study. Mutations of the p53 gene have been reported in different human tumors and has been linked to high tumor grade differentiation, estrogen and progesterone receptor negative status, or HER2 status. BCL-2 protein, on the other hand, is encoded by the BCL-2 gene and plays an anti-apoptotic role by inhibiting cell death, which results in prolonged cell survival. BCL-2 is overexpressed in many cancers, including breast cancer as observed in our study, and contributes to tumor initiation, progression, and resistance to therapy^17,26,32–34^. However, the antagonizing role (negative correlation) between p53 and BCL-2 observed in the current study could be understood through a mechanistic study involving controlling the inhibition of cell proliferation and apoptosis^35^.

Many studies have confirmed the antagonistic expressions among the two interesting biomarkers, p53 and BCL-2. For example, Ruth M. Kluck *et al*.^36^ and Subrata Haldar *et al*.^33^ confirmed the significant correlation between BCL-2 and p53. They stipulated that the level of BCL-2 in cells is regulated by the p53 protein on the principle of feedback. Further mechanistic studies on the roles of p53 and BCL-2 signaling pathways in BC are thus warranted. Regarding the positive correlation between BCL-2 and Ki-67, most of the studies have shown the correlation between BCL-2 and hormonal receptors expression in contrast to Ki-67. Shapochka *et al*.^26^ affirmed in their investigation that the level of tumor proliferation was inversely correlated with expression of hormonal receptors and BCL-2. Nevertheless, this theory needs further research to support it. Meanwhile, this current study did not cover the assessment of hormonal receptors (PR and ER) and HER-2 and association with our prognostic biomarkers or patients’ survival rates, which could have given us more information on patients’ treatment prognosis and/or survival. Further, bigger association studies, involving our studied markers herein (p53, BCL-2 and Ki-67), PR, ER and HER2 are thus warranted, to elucidate their association with BC treatment outcome and/or prognosis in Tanzanian breast cancer patients.

This current study did not include a large number of samples and specific assessment of other molecular subtypes (ER, PR and HER2) that may be associated with p53, Bcl-2 and Ki-67, primarily due to lack of data as reported before. Numerous files were neither labeled as archived nor found in the information system, meaning that the cancer registry system and availability of databases for cancer patients at the MNH and Tanzania still need much improvement. Yet, we still believe that the current results are important for further research work on understanding the possible diagnostic and prognostic functions of the studied biomarkers.

Conclusively, this study has found that Ki-67, p53 and BCL-2 were highly associated with some reported clinical-histopathological features and poor outcomes. The fact that decision-making regarding adequate treatment of patients with BC is based on hormonal receptors, and limited research has been done recently at the national level, it is our hope that advanced studies involving adequately powered samples and other molecular markers, including ER, PR and Her-2, can further be conducted in order to individualize therapeutic schemes without delay.

## Materials and Methods

### Patients and samples collection

**Figure 5:**
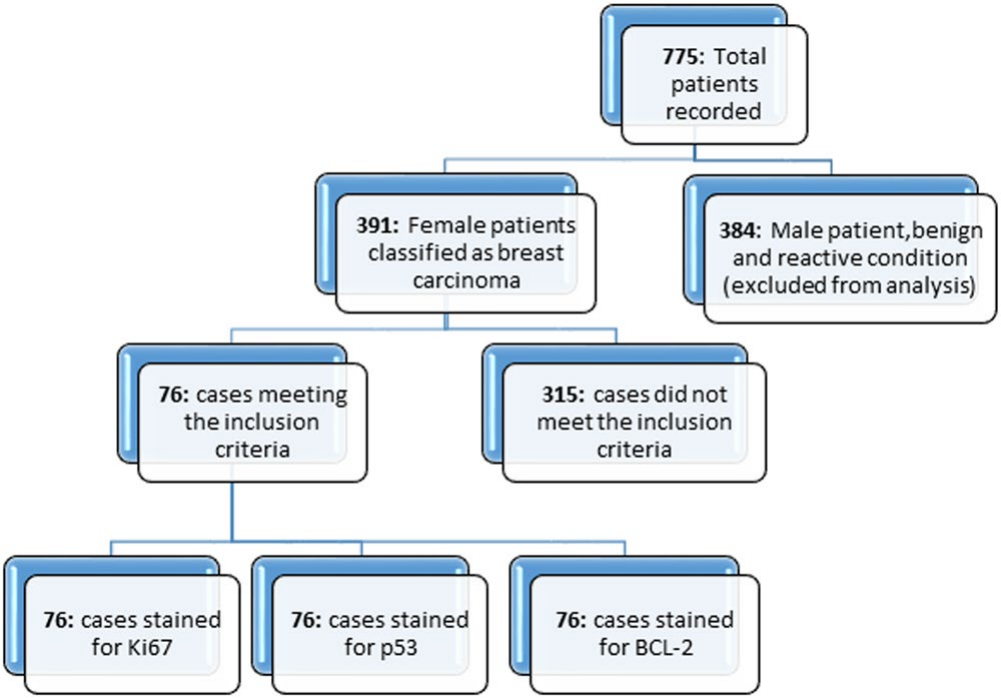
Patient recruitment flow diagram showing distribution of patients and biomarkers categories across different pathological characteristics.

This was a retrospective chart review study that was conducted among females with confirmed breast carcinoma and full clinical histopathological information at the Muhimbili National (MNH) Hospital in Tanzania between 2016 and 2017. Patients were self-presented or referred from different hospitals to the Histopathology Unit or general surgery between December 2016 and December 2017. The target population involved women with confirmed diagnosis of breast carcinoma at the Central Pathology laboratory (CPL). Patients’ clinical and pathological information was acquired from cancer registries. Tissue blocks and Hematoxylin and Eosin (H&E) slides were retrieved from archives for reviewing and diagnosis confirmation. Sorting of slides and retrieval of patient’s information from the cancer registries involved a total of 775 patients. 76 out of 391 cases qualified for downstream analysis of selected biomarkers (Ki-67, p53 and BCL-2) via IHC. The eligible samples were based on breast tissue biopsy (by mastectomy, modified radical mastectomy (MRM), breast excision etc.) with confirmed breast carcinoma, clinical-histopathological information and good morphology. In addition, selected patients were considered to have primary tumor without neo-adjuvant treatment. The exclusive criteria involved 384 patients who were either male patients with breast cancer, patients with a benign condition, patients with secondary BC, missing block tissues and/or those lacking clinical-histopathological information (Fig. 5).

### Immunohistochemistry (IHC) assays

Tissue sections of 3μm, cut on a microtome for IHC staining, were obtained from the retrieved paraffin embedded blocks. Paraffin sections were left in floatation water bath at 45 °C, to eliminate wrinkles and distortions of tissue, followed then by tissue bonding on a glass and placement in a hot plate at 60 °C for 24 hours, to get rid of the wax before undertaking Ki-67, p53, and BCL-2 staining. Moreover, Monoclonal Mouse antibodies for Ki-67 (Dako Monoclonal Mouse Anti-Human, MIB-1 ready to use), p53 (Dako Monoclonal Mouse Anti-Human, DO-7 ready to use) and BCL-2 (Dako Monoclonal Mouse Anti-Human, 124), Target Retrieval Solution High pH(50x), Peroxidase-Blocking Reagent(ready-to-use), HRP (ready-to-use), DAB + Chromogen, Substrate Buffer, as well as Wash Buffer (20x) were all provided by Labulax Supplies Limited (Nairobi, Kenya).

Furthermore, the staining process was firstly based on moisture chamber preparation, followed by application of 10 min absolute xylene for tissue de-paraffinization, 10 dips of tissue hydration with 100–95% alcohol and washing slides with water for almost 5 min (minutes). The next step was characterized by application of diluted low pH Target Retrieval Solution (50x) under pressure cooker for 13 min and washing slides with diluted Wash Buffer (20x) for 5 min after each application of the following; Peroxidase-Blocking Reagent to the section for 15 min, Ki-67, p53 protein and BCL-2 oncoprotein as primary antibodies for 30 min, and HRP (Dextran couple with peroxidase molecules and goat secondary antibody) for 30 min (3 times washing buffer each). In the same way, the application of DAB (diaminobenzidine) + Chromogen reagent for 10 min were performed on the slides through dilution of 1drop of DAB concentrated into 1 ml substrate chromogen reagent, followed by water washing of slides. Slides were then counterstained with hematoxylin for 17 dips, followed by washing slides with water for 5 min and dehydration in 95% to absolute alcohol, then cleared the slides with xylene before mounting with tissue tek-coverslip machine. Finally, false positive was eluded by a positive control test.

### Histopathology classification

The histopathological classification of breast cancer was defined in accordance with the worldwide TNM (Tumor, Node, and Metastases) classification of divers’ cancer by WHO 2003^29^. Additionally, tumor grade was performed under the Nottingham grading system. The sub-score from 1 to 3 was assigned on each of the following features: the amount of tubules formation, nuclear pleomorphism, and mitotic index. Thus, grade III of cancer was assigned to any patients with a Nottingham score of 8 or 9. Grade II referred to Nottingham scores of 6 and 7, while grade I referred to Nottingham scores of 3, 4, and 5.

### IHC staining assessment and evaluation

The cutoff point for IHC staining of Ki-67, p53, and BCL-2 varies from literature and studies^23,32,37,38^. Thus, microscopic evaluation of the slide sections in this study was done through cell counting by recording cells proliferation, as well as the number and intensity of positively stained cells for Ki-67, p53, and BCL-2. Positive slides were considered from 10 to more than 14% of the cells stained, with a score assessment of 2+ (moderate intensity/moderate proliferation) and 3+ (high intensity/high proliferation). In contrast, negative results were observed from the section slides in which less than 10–14% of cells were stained, following a score of 0–1+ (absence of cells staining or low intensity/low proliferation). The IHC evaluation for each studied biomarker was thus conducted as per following Table 5.

**Table 5:** Microscopic evaluation of IHC staining.

IHC assessment	**Low biomarkers expression** (**negative**)	**High biomarkers expression** (**positive**)
	The high intensity of cells staining with low proliferation. Low intensity of cells staining with high proliferation. An absence of cells staining.	High to moderate intensity of cells staining with high to moderate cells proliferation.

### Data handling and statistical analysis

Clinico-histopathological characteristic as well as biomarkers expression data were entered into structured check-list and Excel spreadsheet software. Data was cleaned and manipulated several times and analyzed using both the R software^39^ and SPSS software version 23. A Fisher’s exact test was performed to assess the relationship between biomarkers expression and the clinico-histopathological response. A multinomial linear regression was performed to identify the association between Ki-67, p53 and BCL-2 markers and all clinical histopathological variables. Correlation test was used to evaluate the degree of association among variables. Statistical significance was taken at p-value < 0.05^40^.

### Ethics approval and consent to participate

Ethical clearance of this study was sought and obtained from the National Institute for Medical Research (NIMRI) with reference number NIMRI/HQ/R.8a/Vol. IX/2707. Method and permission to conduct the research study was sought and accepted by the relevant authority of Muhimbili National Hospital. Consent of participation was not applicable. Informed consent before enrolling this study was declared not needed by the national ethical committee.

## Data Availability

The datasets generated during and/or analysed during the current study are not publicly available due to strict confidentiality requirements for patients, but are available from the corresponding author on reasonable request.
